# Supplementary material for: Are carbon emissions trading and green financial instruments synergistic? -Comprehensive quantitative research based on content analysis
Source: PLoS One. 2024 Mar 7;19(3):e0298601. doi: 10.1371/journal.pone.0298601 (PMC10919601; doi:10.1371/journal.pone.0298601)
Supplement: S1 Data — (ZIP) [file pone.0298601.s001.zip › Data/地级市Z_F工作报告.zip!/╜¡╦╒/╨∞╓▌╩╨/└┤╫╘╣1⁄2╓┌║┼í╛┬φ┐╦╩2╛▌═°í┐_2017.docx]

各位代表：

现在，我代表市人民政府向大会作工作报告，请予审议，并请市政协各位委员和其他列席人员提出意见。

一、过去五年工作回顾

2012年以来，我们在市委的坚强领导下，全面落实党的十八大和十八届三中、四中、五中、六中全会精神，主动适应把握引领新常态，认真践行五大发展理念，与时俱进抓好各项重点工作，较好地完成了市十五届人大一次会议确定的各项目标任务。（一）五年来，综合实力不断增强。地区生产总值连跨两个千亿元台阶，达5808亿元，年均增长10.6%。一般公共预算收入连跨两个百亿元台阶，达516亿元，是2011年的1.6倍。固定资产投资、社会消费品零售总额分别达4797亿元和2659亿元，年均增长16.9%、18.4%，总量实现翻番。进出口、出口总额分别达414亿元和348亿元。（二）五年来，产业转型实现突破。产业结构由9.4:50.1:40.5变为9.3:43.3:47.4，实现了“二三一”到“三二一”的重大转变。工业总产值达1.4万亿元，装备制造、食品及农副产品加工、煤盐化工等8个产业规模均超千亿元，服务业增加值占比提高6.9个百分点，农业综合生产能力持续提升。科技创新支撑体系初具规模，全社会研发投入占比达2%左右，高新技术产业规模达5177亿元。相继获批国家智慧城市试点市、电子商务示范市、信息消费示范市。（三）五年来，城乡面貌深刻变化。累计实施城建重点工程790项，458项基础设施和功能性项目竣工使用，各项设施配套日趋完善，中心城市框架全面拉开、功能大幅增强、形象不断提升。中等城市、中心镇、管理示范镇建设和村庄环境整治卓有成效。全市城镇化率达62.4%，提高6.9个百分点。（四）五年来，生态建设成效明显。美丽徐州建设“五大行动计划”深入实施，节能减排超额完成省定任务，空气和水环境质量持续提升。先后对18万亩采煤塌陷地、3.4万亩工矿废弃地、43处采石宕口实施生态修复。新增造林65.5万亩，林木覆盖率达30.3%，建成区绿化覆盖率达43.7%。跻身国家节能减排财政政策综合示范市、循环经济示范市和全国首批水生态文明建设试点市。先后获得国家森林城市、卫生城市等称号，成为全国首批生态园林城市，赢得“一城青山半城湖”的美誉。（五）五年来，群众生活显著改善。城乡居民收入分别达28421元和15274元，分别是2011年的1.6倍和1.7倍，年均增长9.8%和11%。年人均纯收入4000元以下的87.7万贫困人口如期脱贫。新增就业连续五年超10万人。城乡一体的居民基本养老保险、医疗和大病保险制度基本建立，教育、卫生、文化、养老资源总量持续增加、结构趋于优化，县域义务教育基本均衡实现全覆盖。成功举办第18届省运会。（六）五年来，政府建设全面加强。精心组织开展党的群众路线教育实践活动、“三严三实”专题教育和“两学一做”学习教育。法治政府建设步伐加快，编制完成“五张清单”，取消和下放市级行政审批事项381项。重点领域和关键环节体制机制改革深入推进。中央八项规定精神和省、市委十项规定得到有效落实，反腐败斗争深入开展，政风行风进一步好转。

各位代表！2016年是本届政府任期的最后一年，也是“十三五”开局之年。一年里，全市经济社会发展稳中有进、稳中向好，地区生产总值增长8.2%，一般公共预算收入同口径增长6.1%，进出口、出口、社会消费品零售总额、城乡居民收入增速均居全省首位，成功实现“十三五”发展良好开局。

（一）坚持产业发展的鲜明导向。我们坚持把产业发展作为立市之本、强市之基，旗帜鲜明地提出了“工业立市、产业强市”的指导思想，聚焦建设“一中心一基地一高地”，加速构建“6+6”先进制造业、“333”现代服务业、现代农业产业体系。一是产业结构调整有力有效。规模以上工业增加值达3010亿元、增长9.8%，新兴产业产值增长13.4%，轻工业产值增速高于重工业2.7个百分点，高耗能行业产值增速低于规模以上工业4.1个百分点。服务业增加值增长9.1%，占比同比提高1.2个百分点；我市列入国家“十三五”服务业综合改革试点区域。设施农业新增面积10.6万亩，总量和占比保持全省领先，粮食生产实现“十三连丰”。单位GDP能耗下降5%左右。二是产业投资力度明显加大。全市固定资产投资增长12.5%，其中制造业、工业技改投资分别增长16.7%和26.2%。在建亿元以上产业（不含房地产）项目951个、同比增加378个，完成投资2322.6亿元、增长32.7%。徐工重型汽车、协鑫铸锭切片等项目建成投产，铜山万达广场、徐州综合物流园等项目开业运营，苏宁广场、三胞广场等项目主体竣工，光刻产业科技园、瑞典宜家、英国福来德百货等高端项目签约落地，华为、微软、软通动力、阿里巴巴等一批ICT企业进驻徐州。三是经济运行质效不断提升。全市规模以上工业企业利润、利税总额分别增长13.3%和11.9%。新登记企业3.7万个，增长46.5%，注册资金增长68.8%。存贷款余额分别达5640亿元和3630亿元，增长18%和17.6%，当年新增存款893亿元、创历史新高，新增贷款560亿元、同比多增215亿元。

（二）高水平建设区域性中心城市。明确提出打造“五个城市”及“四个中心”的城市定位，着力构建中心城市“2+6+15”空间布局和五级新型城镇体系，不断增强城市集聚辐射带动力。一是城市综合功能建设加快。完成徐州都市圈规划修编和城乡统筹、重要功能片区、镇村布局优化、新型农村社区等专项规划编制。190项城建重点工程开工172项，开工率达91%，竣工76项。郑徐客专开通运营，三环北路高架快速路建成通车，徐济高速全线开通，轨道交通1、2、3号线全部开工建设，空军徐州机场迁建以及徐宿淮盐铁路、徐明高速等工程进展顺利。完成56.7公里城市道路改造，新增停车泊位3.2万个、更新泊位4600个。中心医院新城区分院、二院开发区分院投入使用，第一医院迁建、北区股份制医院建设有序推进。骆马湖水源地及徐庄水厂建成使用，结束了市区供水长期采用地下水的历史。启动市区棚户区改造579.8万平方米。完成73个老旧小区环境综合整治。云龙湖、大龙湖景区分别获批国家5A级旅游景区、省级旅游度假区。二是城市环境面貌日趋改善。文明城市创建深入推进，背街小巷、集贸市场等专项整治成效明显，文明礼仪教育、志愿服务等活动广泛开展，群众文明素质不断提高。“城管+公安”联动执法、环卫保洁市场化一体化等城管机制逐步健全，246条道路街巷、22类突出问题专项治理扎实推进。完成387台燃煤锅炉拆除或改燃工作，淘汰黄标车和老旧车辆近3万辆，市区PM2.5平均浓度有所下降。市区黑臭水体整治加快实施，国家考核断面水质达标率100%。成功创建省级生态市和省优秀管理城市，获得中欧绿色和智慧城市奖。以优异成绩荣膺中国人居环境奖。三是新型城镇体系建设有序展开。制订新型城镇化建设实施意见，确立“1530+30+130”城镇体系。5个中等城市产业和人口集聚功能进一步增强，新一轮新型城镇建设试点启动实施。创建省级美丽村庄、康居村庄和村庄保护项目32个，建成市级新农村示范村40个。

（三）全力培育新的经济增长动力。聚焦深化改革、创新驱动、双向开放，加快培植新的增长点，筑牢经济转型提升发展的坚实支撑。一是力推各项改革落地见效。供给侧结构性改革扎实推进，“三去一降一补”取得阶段性成果，关闭矿井12对、压减煤炭产能1044万吨，安置分流职工2.7万人，拆除中频炉炼钢设备21套，淘汰粗钢产能近200万吨，彻底取缔“地条钢”企业5家；商品住房去化周期由15.3个月下降到7.5个月；新增“新三板”上市企业11家、场外挂牌企业154家，利用各类债务融资工具融资274.6亿元；落实减税降费政策，全面推开“营改增”试点，全年降低企业负担55.5亿元；实施六大领域32项补短板工程，134个年度项目完成投资450亿元。持续加大简政放权力度，下放和调整权力事项212项，清理规范中介服务事项12项。积极探索相对集中许可权改革，推行联合评审、并联审批，整合建立市公共资源交易中心等综合便民服务平台。“123”开头的服务专线整合联动顺利完成，5A政务服务信息化系统平台上线运行，“多证合一、一照一码”登记模式和个体工商户“两证整合”全面推行。率先完成国家级、省级综合行政执法体制改革试点任务。金融改革创新力度明显加大，徐州恒鑫金融租赁公司、江苏联徐资产管理公司等相继开业运营，淮海金融资产交易平台获批筹建，我市被评为“中国金融生态城市”。农村土地承包经营权确权登记颁证步伐加快。成为全国水流产权确权试点唯一地级市。二是切实打造创新支撑载体。全力建设淮海科技城、徐州科技创新谷、潘安湖科教创新区等重大创新平台，扎实开展“赢在徐州”创新创业大赛，不断增强创新驱动能力。年内新增国家高新技术企业64家，获批国家级众创空间2家、省级众创空间13家，新认定省大学生创业示范园2个。省级以上孵化器达35家，总面积达100万平方米。高新技术产业产值增长14.9%，专利授权量增长22.4%。徐州安全科技产业园被命名为首个国家安全产业示范园区。“智慧徐州”建设进展加快，淮海科技大数据产业园、徐州智慧信息产业园相继开园，电信淮海经济区数据中心建成使用，全市互联网出口带宽达3200G、当年新增1300G，市县村光缆联网率达100%。三是积极开辟对外开放新空间。成功举办英德、日韩、京沪、十九洽会等重点招商活动，引进5亿元以上内资和3000万美元以上外资重大项目81个，协议利用外资35.1亿美元、增长121%。开放型经济“十大平台”建设成效显著，徐州保税物流中心封关运营，观音机场新开通日本大阪、韩国仁川国际航线，徐新亚国际货运班列开通运营，电子口岸建成并试运行，鼓楼区获批省级跨境电商产业园，新增4家省级开发区和高新区。全市进出口、出口总额分别增长23%和27.5%。服务外包合同额、执行额分别增长9.2倍和12.5倍。我市被认定为省服务外包示范城市。

（四）拉长补齐民生短板。把改善民生作为政府工作的出发点和落脚点，积极构建社会建设“十二大体系”，最大限度让群众共享发展成果。一是民生实事得到有效落实。8大类72项实事项目总体完成年度任务。城乡居民收入分别增长8.4%和9.2%。新一轮扶贫开发扎实推进，26.2万户、68.2万名低收入人口全部完成建档立卡，20.2万名低收入人口实现脱贫，105个经济薄弱村综合服务中心工程基本完工。投资86.5亿元的城乡供水一体化工程全面启动建设，127万农村居民吃上安全放心水。新建农村公路1164公里，新建改建农村道路桥梁164座，疏浚县乡河道766.5公里，整治村庄河塘1773条。二是就业和保障水平不断提升。新增城镇就业11.9万人、失业人员再就业10万人、农村劳动力转移5.7万人，城镇登记失业率控制在2%。全民参保登记计划深入实施，主要险种参保覆盖率均超过95%。城乡低保标准自然增长、重特大疾病医疗救助、困境儿童分类保障、困难残疾人生活补贴等机制不断完善。三是各项社会事业均衡发展。“基础教育学南通”活动深入开展，“双提升”工程有力推进，新建改建中小学36所、幼儿园111所，我市被确立为“全国基础教育课程改革实验区”。公立医院综合改革、分级诊疗制度稳步推进，三级医院增至16家，三级甲等医院达7家，国家和省级临床重点专科达64个。“舞动汉风”工程、“文润彭城”活动扎实开展。第21届市运会、首届中国·徐州国际武术大赛成功举办。徐州籍健儿在里约奥运会上获得1金、2铜的优异成绩。

（五）全面提升社会治理能力。环境保护、安全生产、食品药品安全、信访稳定“四项督查”深入开展。社会稳定风险评估、矛盾纠纷化解、社会治安防控和应急处置等工作不断加强。“好人文化”建设进一步深化，新入选“中国好人”8名，成功承办全国“最美家庭讲好家训”万场巡讲工作经验交流会。城乡和谐社区创建达标率分别达92%和85%，国家级、省级和谐社区示范单位分别达11个和507个。刑事案件立案数同比下降6.3%。安全生产事故总量和死亡人数继续实现双下降。人民调解“徐州模式”更加完善。社会信用体系建设扎实推进。获评“全国法治宣传教育先进城市”。全国双拥模范城创建实现“八连冠”。人防、国防动员、民族宗教、档案、外事、侨务、台湾事务、审计、统计、物价、新闻出版、机关事务管理、住房公积金管理、无线电管理、淮塔管理、防震减灾、气象、老龄、青少年、妇女儿童、关心下一代等各项事业都取得了新进步。

（六）完善政府工作落实机制。制定进一步健全完善政府工作高效运行机制的意见，积极创新机制、健全制度、优化流程，切实提高政府组织经济工作、破解复杂难题、推动工作落实的能力。一是创新政策激励机制。成功争取省委、省政府出台《关于全面振兴徐州老工业基地，加快建设“一带一路”重要节点城市的意见》，出台扶持现代产业发展若干政策，由财政安排200亿元的奖励和引导基金支持产业发展，开展“徐州经济年度人物”和“十佳优秀企业家”评选，全面优化政策环境。二是创新考核导向机制。推行县（市）区重点经济工作、条线部门创新奖、干部作风绩效“三大考核”，进一步突出考核重点，大幅精简考核事项，把广大干部的注意力、主要精力引导到产业发展、创新实干上来。三是创新工作推进机制。构建组织协调督查三大体系，建立市政府每周工作例会、重点工作定期调度、“三个一”工程重大项目包挂以及督查督办等制度，实现了政府工作全过程管理、高效率运转。

回顾五年来的政府工作，这些成绩的取得，是省委、省政府和市委正确领导的结果，是全市人民团结拼搏的结果，也是社会各界共同努力的结果。在此，我代表市人民政府，向全市各族人民群众，向人民解放军驻徐部队、武警官兵和公安民警，向各民主党派、工商联、无党派人士和人民团体，向支持徐州建设的香港特别行政区同胞、澳门特别行政区同胞、台湾同胞、海外侨胞和各界人士，向投身徐州经济社会发展的国内外投资者和企业家，表示衷心的感谢和崇高的敬意！

我们也清醒地认识到，当前我市经济社会发展仍然面临一些突出问题：经济下行压力依然较大，地区生产总值、固定资产投资增速没有达到年度预期，传统动力支撑作用减弱，部分企业生产经营困难；科技创新能力仍然不强，开放型经济短板亟需补齐，淘汰落后产能、推动产业转型升级任重道远；中心城市功能有待进一步提升，城乡二元结构较为明显；生态环境尚未根本好转，大气质量改善形势严峻，节能减排、达标排放任务艰巨；受政策调整影响，一般公共预算收入减收明显，财政收支平衡矛盾加剧；居民增收压力加大，公共服务水平需要进一步提高；一些干部的责任意识、担当意识和服务意识不强，有些部门办事效率不高，作风建设和反腐倡廉长效机制仍需完善等等。对这些问题我们将高度重视，采取有力措施，切实加以解决。

二、今后五年的奋斗目标

今后五年，是徐州全面建成小康社会的决胜期，是老工业基地全面振兴转型的攻坚期。徐州新一轮发展面临着三个非常现实的状况：一是徐州作为重要的交通枢纽、区域性中心城市的区位优势，如何转化为现实的发展优势？二是在经济发展新常态下，徐州的发展是跟随发达地区的脚步走，还是摆脱传统发展路径的依赖，在新兴产业、创新型经济发展上追求突破，走出具有自己特色的绿色、生态、创新发展新路子？三是在当前经济全球化的大潮中，生产要素流动重组日趋活跃，科技创新日新月异，颠覆性商业模式不断涌现，国内部分大城市受高房价等因素影响商业运行成本不断攀高，形成了生产要素的挤出、溢出效应，我们如何抓住机遇，实现生产要素的快速集聚，抢占发展的先机？对此，我们要准确把握外部环境、内在条件、阶段特征和动力支撑的新变化，立足优势谋发展，发挥优势求突破，打破产业梯次转移、跟随发展的思维定式，摆脱对传统路径的依赖，更高层次上吸纳要素资源、更大范围内汇聚发展能量、更大力度推进创新型经济发展，努力在新常态下实现弯道超越，谱写好徐州发展的崭新篇章。

今后五年政府工作的总体要求是：高举中国特色社会主义伟大旗帜，深入学习贯彻习近平总书记系列重要讲话精神，紧紧围绕“五位一体”总体布局和“四个全面”战略布局，自觉践行新发展理念，坚持以人民为中心，发挥“三个作用”，推进“两聚一高”，建设“强富美高”新徐州，全力打造淮海经济区中心城市、全国老工业基地振兴示范城市、“一带一路”重要节点城市、全国重要的综合交通枢纽城市、宜业宜居的民生幸福城市。

主要预期目标是：

——综合实力显著增强。经济增长高于全国全省平均水平，地区生产总值、一般公共预算收入力争达到8000亿元和800亿元，以高新技术产业为主导、先进制造业为支撑、服务经济为主体、现代农业为基础的现代产业体系初步形成，经济总量在淮海经济区的领先优势进一步扩大。

——民生福祉显著增加。城乡居民收入分别达45000元和25000元，脱贫致富奔小康工程圆满收官，社会建设水平不断提高，城镇社区形成医疗、社保、文化、健身、养老等15分钟便民服务圈，城乡居民享有更好的教育、更稳定的就业、更满意的收入、更可靠的社会保障、更高水平的医疗卫生服务、更舒适的居住条件、更优美的环境。

——科技创新能力显著强化。研发投入占比达2.5%以上，高新技术产业产值占比达45%以上，主要创新指标达到全国中上水平，进入国家创新型试点城市行列。区域性产业研发集聚、产业化发展集聚、创新产业孵化集聚、高层次人才集聚态势基本形成。

——城乡发展水平显著提高。中心城市功能、品质、能级显著提升，城市“首位度”显著提高，淮海经济区中心城市地位进一步确立。新型城镇化和城乡发展一体化扎实推进，城乡、区域发展优势互补、融合互动格局基本形成，城镇化率提高到68%以上。

——双向开放领域显著拓展。经济国际化水平不断提升，成为新亚欧大陆桥沿线开放高地。利用外资规模质量全面提高，进出口总额实现翻番，各级各类开发区的功能、承载能力进一步增强，在全国全省的地位、在全市的经济份额显著提高。

——人居生态环境显著优化。环境保护、生态治理取得重大成果，空气质量优良天数比例达70%以上，地表水水质优良比例达80%以上，建成区绿化覆盖率达44%以上，让徐州的天更蓝、地更绿、水更清、空气更清新。

——社会文明程度显著提升。中国梦和社会主义核心价值观更加深入人心，群众思想道德素质、科学文化素质、健康素质明显提高，精神文化需求得到更好满足。文化事业和文化产业繁荣发展，建成全国文明城市。

——社会治理能力显著进步。法治政府加快构建，社会治理体系和治理能力现代化建设取得重大进展，社会更加稳定和谐，法治建设满意度、人民群众安全感位居全省前列。

三、2017年主要工作任务

今年是实施“十三五”规划的重要一年，也是推进供给侧结构性改革的深化之年。全市经济社会发展主要预期目标是：地区生产总值增长8.5%左右；一般公共预算收入同口径增长5%左右；固定资产投资增长12%左右；社会消费品零售总额增长12%以上；进出口总额增长10%以上；实际到账注册外资增长10%以上；城乡居民人均可支配收入增长快于GDP增长；研发投入占比力争达2.2%；居民消费价格涨幅控制在3%左右；城镇登记失业率控制在4%以内；节能减排和大气、水环境质量确保完成省定任务。新的一年里，要重点抓好以下几个方面的工作：

（一）做强产业经济，夯实全面振兴转型、可持续发展的坚实基础。牢牢抓住产业发展这个根本，全力落实建设“一中心一基地一高地”和发展现代农业四大任务，推动经济持续平稳增长，以产业振兴带动老工业基地“凤凰涅槃”。

加快培育壮大主导产业。坚持改造提升传统产业和培育壮大新兴产业并重，以打造一批在世界有知名度、在全国有地位的主导产业为目标，着力构建具有全球知名度的装备与智能制造基地、新能源产业基地；力争集成电路与ICT、医药医疗与健康产业发展取得重大突破，加快形成国内领先优势；推动食品、煤盐化工、冶金、建材等产业延伸链条、壮大规模、提升竞争力，加快向中高端发展。紧盯新兴产业发展趋势，坚持“互联网+”和“+互联网”并举，促进“四新经济”茁壮成长。紧紧抓住项目龙头，加大投入力度，重点实施徐工重型矿用机械、必康医药、鑫华半导体级多晶硅、软通动力创新平台、华为淮海大数据中心等重大产业项目，培育和扶持一批新兴产业集群和龙头企业，推动主导产业不断壮大、加快优化升级。

大力发展现代服务业。全力推动国家级服务业综合改革试点，进一步完善“333”现代服务业体系，打造中心商圈、高铁商务区、空港经济等功能板块，抓好君太百货、金鹰二期、月星环球港等重大服务业项目建设，力争年内服务业占比再提高1个百分点。抓好北三环物流集聚带及苏山铁路物流园、亿吨大港物流园、林安物流园、潘塘物流园等物流基地建设，办好中国国际物流发展大会，扩大区域物流中心辐射范围。积极发展全域旅游，提升云龙湖5A级景区建设管理运营水平，建设更多影响力大、吸引力强的精品景区。加快申报国家金融支持服务业综合改革试验区，培育和完善多层次资本市场，大力发展证券、期货、创投、风投、天使投资等金融业态。加快发展云计算、大数据及平台经济、总部经济、楼宇经济、会展经济，培育科技信息、旅游文化、健康养老、教育培训等消费热点。举办国际服务外包大会，促进服务外包产业加速成长。

推动现代农业提质增效。坚持把“三农”工作摆在重要位置，大力推进农业供给侧结构性改革，推动农业增效、农民增收、农村增绿。构建现代农业“483”产业体系，做强优质粮食、设施蔬菜、高效林果、生态畜牧等四大主导产业，做精大蒜、食用菌等八大特色产业，做优农产品加工、休闲农业、智慧农业等三大融合产业，打造黄河故道现代农业、城郊都市农业、一二三产业融合发展三大示范带，创建国家农业可持续发展试验示范区。推广农田托管、土地入股、联耕联种等生产方式，发展多种形式的适度规模经营，培育新型农业经营主体，促进“互联网+”现代农业发展。继续实施设施农业“2020”工程，抓好现代农业示范园区、农产品加工集中区、粮食物流园区建设。大力推进黄河故道综合整治，严格耕地保护，加强永久性基本农田、高标准农田建设和粮食高产示范创建，实施重大水利工程和节水型农田水利项目，推进粮食生产全程机械化，保护和提高粮食综合生产能力。狠抓农产品标准化生产，构建农产品质量安全追溯体系，培育更多的绿色优质农产品品牌。

充分激发企业市场主体活力。在全社会形成崇尚发展、尊重企业家的浓厚氛围，建立新型“亲”、“清”政商关系，营造一流的营商环境，促进企业健康发展。健全包挂联系重点企业制度，深化政银企对接，加大要素协调力度，及时帮助企业排忧解难。发挥产业引导基金的撬动作用，抓好项目与基金的对接，带动更多社会资本投入实体经济。支持企业发行各类债券，扶持有条件的企业在境内外上市，推动股权及债券融资扩围，拓展多元化融资渠道，缓解企业融资难问题。树立质量第一的强烈意识，开展质量品牌提升行动，引导企业发扬“工匠精神”，培育更多“百年老店”，打响“徐州制造”品牌。

（二）聚力创新驱动，构建区域领先、体系健全的创新高地。创新是引领发展的第一动力，必须摆在发展全局的核心位置。要积极推动以科技创新为核心的全面创新，加快形成以创新为主要引领的经济体系和发展模式。

建设一流产业科技创新载体。加快淮海科技城规划建设，完善基础设施、组织机构和运营平台，打造集众创空间、产业研发加速器、科技成果孵化器、科技金融集聚区等创新容器为一体的孵化基地。大力推进科技创新谷建设，重点围绕主导产业和战略性新兴产业，集聚配置全球优势创新资源，构建区域性高新技术产业发展高端平台。高标准规划建设潘安湖科教创新区，加快江苏师大科文学院迁建，打造产学研一体、学城融合、智慧生态的综合型科教创新基地。抓好产业技术研究院建设，建立健全配套服务功能模块，形成以产业前瞻性技术、共性技术和关键技术为研究重点的“1+N”式产业研究集群。

构建开放式产业科技创新网络。强化企业创新主体地位，推动企业研发平台提档升级，支持徐工、中能等行业龙头企业建设重点实验室，鼓励大中型企业创建国家工程实验室、工程技术研究中心等创新平台。提升高新区产业研发及产业化发展水平，支持有条件的地区创建省级高新区。安排市校合作专项资金，鼓励校地联合研发关键技术、转化科技成果、开展引才育才、建立创新创业载体，积极引进国内重点高校在徐建设技术转移中心。推进 “互联网+”与传统创新创业载体融合，发展线上线下相结合的新型众创空间。加快创建国家知识产权示范城市。

吸引创新要素加速集聚。深入实施“彭城英才”计划，全力集聚一批领军人才和团队，以更加优惠的政策、更加优质的服务留住人才、激励人才发挥作用，力争年内引进20名左右“诺贝尔奖”获得者、“两院”及外籍院士、“千人计划”等国家级领军人才，引进200名左右海内外高层次人才、2000名左右硕士博士来徐就业创业。用好科技企业贷款风险补偿基金，探索建立科技与金融服务中心，推动徐州高新区科技金融合作创新示范区建设。继续实施“创业徐州”行动计划，提升“赢在徐州”创新创业大赛影响力。

（三）聚焦富民举措，不断提高人民群众获得感和幸福感。坚持民生为本，以民之所望为施政所向，进一步提高居民收入、社会保障和公共服务水平，努力让徐州百姓过上更加美好的生活。

抓好富民增收和扶贫开发。将富民作为最大的民生实事，继续实施八项富民工程，加快发展富民产业，多方式挖掘增收潜力、提高群众收入，让城乡居民的钱袋子尽快鼓起来。大力发展民营经济，降低准入门槛，打造民营经济集聚区，出台优惠政策，解决办事难、贷款难等突出问题，把民营经济发展为徐州的主流经济、百姓致富的重要渠道。完善企业职工工资正常增长机制，高度重视农民工工资支付保障问题，推动居民工资性收入动态增长。深入推进新一轮扶贫开发，提高精准扶贫、精准脱贫水平，年内确保17万低收入人口脱贫、68个经济薄弱村实现新“八有”目标。强化产业开发、就业帮扶、转移支付、政策兜底，抓好丰县湖西片区、黄墩湖滞洪区、黄河故道沿线连片帮扶。

提升就业和社会保障水平。落实更加积极的就业扶持政策，重点解决好高校毕业生、城镇零就业家庭、最低生活保障家庭、去产能转岗下岗职工等群体的就业问题，努力实现更加充分、更高质量的就业。深入推行全民参保计划，抓好机关事业单位养老保险制度改革，加快推进居民医保和新农合制度整合，建立基本长期照顾护理保险制度，稳步提高基本医疗保险水平，不断扩大住房公积金制度覆盖面。健全社会救助体系，完善临时救助制度，健全城乡居民低保标准、城市“三无老人”和孤儿供养标准自然增长机制。进一步扩大医疗救助范围，建立困难群众医疗救助新机制，有效遏制因病致贫、因病返贫现象。推进社会福利服务，继续开展安康关爱行动，实施困难残疾人生活补贴和重度残疾人护理补贴制度，抓好残疾人之家建设，建立困境儿童分类保障机制。建设定销房300万平方米，建成公租房1000套。

优化公共服务供给。大力推进公共服务的标准化、均等化、特色化建设。深化“基础教育学南通”活动，努力提高基础教育的质量和水平。新建、改扩建幼儿园184所、小学79所、初中38所，加固重建校舍48万平方米。加大无证幼儿园清理规范力度，创建义务教育现代化学校150所，加快三星级以上高中建设，推进职业教育创新发展和职业院校提档升级。深化综合医改，大力度引进、培养高层次医疗人才，重视医疗设备、技术更新投入，不断提升医疗服务水平；进一步抓好分级诊疗制度建设，促进优质医疗资源均衡分布，提升县级医院和基层医疗卫生机构服务的水平和能力。加强和完善计划生育服务管理，促进人口长期均衡发展。健全养老服务体系，抓好养老机构建设，提升社区居家养老服务品质和覆盖面，加快推进医养融合发展。积极发展老龄事业，办好老年大学。举办国际马拉松赛和国际武术大赛，促进全民健身、竞技体育和体育产业协调发展。深入实施“舞动汉风”工程，推进公共文化服务设施网络达标建设，加强文化遗产传承保护，实施精准文化惠民项目，开展全民阅读活动，打造“书香徐州”。

深化社会治理创新。持续加强法治徐州、平安徐州建设，实施群防群治“守护工程”，构建大数据支撑下的立体化、信息化社会治安防控体系。加强和改进信访稳定工作，完善矛盾纠纷多元化解机制，把各类矛盾化解于基层、解决在萌芽状态。做好政社互动和社区减负。开展“七五”普法教育，抓好公共法律服务惠民。更大力度抓好社会诚信体系建设，让守信者受益、失信者难行。巩固双拥创建成果，促进军民深度融合发展。完善和落实安全生产责任、管理制度和考核机制，狠抓隐患排查整治，坚决遏制较大及以上安全事故发生。严厉打击食品药品安全违法行为，完善应急体系建设，提高公共安全和防灾减灾能力。

各位代表，办好民生实事是促进民生改善、增加民生福祉的重要切入点。今年我们围绕民生需求，充分听取群众意见，安排了与群众生活密切相关的70件民生实事，涵盖教育提质、富民增收、社会保障、健康惠民、安居宜居、养老服务、公共交通、文化体育、生态环境、公共安全十个方面。办好这些实事，是市政府对全市人民作出的庄严承诺。我们将高度重视，强化协调调度，狠抓工作推进，确保把实事抓好，向人民交出一份满意的答卷！

（四）提升城市品质，构建富有魅力、宜业宜居的区域性中心城市。以打造淮海经济区中心城市为引领，加快城市现代化、新型城镇化和城乡一体化步伐，让我们的城市有神有魂、有个性有品位、有魅力有活力，实现由“靓丽徐州”到“品质徐州”的提升。

加快完善城市功能。做好新一轮城市总体规划和徐州都市圈规划修编，科学谋划徐州2049空间战略布局，提高专项规划编制水平。启动黄河故道沿岸生态观光带建设，加快推进圣戈班、翠屏山、空军机场等重点区域搬迁改造，高标准抓好150项城建重点工程建设。提升综合交通功能，抓好轨道交通1、2、3号线和迎宾大道、城东大道快速路等项目建设，加快推进徐连客专、徐宿淮盐铁路徐州段、外环公路等重点交通工程，推动高速铁路、干线铁路、高速公路直达全国主要城市群，完成观音机场二期扩建，强化与周边城市基础设施互联互通。提升物流中转功能，主动参与亚欧国际陆桥物流大通道建设，构筑公铁、铁水、河江海、空铁等联运体系，加快把徐州建设成为区域多式联运中心和国家级综合物流枢纽；规划建设进出口商品交易中心，打造区域性国际会展基地。提升金融服务功能，抓好金融平台建设，吸引各类金融机构来徐设立分支机构，充分发挥金融在区域经济发展中的引领支撑作用，打造区域性金融服务中心。提升信息平台功能，加快互联网国际通信专用通道建设，年底互联网出口带宽达到3500G，打造“线上版”网络信息枢纽；提升基础设施承载功能，大力推进城乡供水一体化工程，统筹抓好城市防洪、排水及海绵城市、综合管廊建设。实施1526万平方米棚户区（城中村）改造，抓好432个老旧小区综合整治和长效管理。

促进城市品质全面提升。积极弘扬徐州特色地域文化和优良传统文化，开展好群众性精神文明建设，充分彰显楚韵汉风、南秀北雄的城市特质和重情重义、敢想敢干的人文精神。启动建设一批城市文化、社会事业等功能性项目，加快打造高效便利的文化、生活服务体系。以全国文明城市创建、全国卫生城市复检为抓手，强化市容环境综合整治，有效解决“脏乱差”问题，促进市民摒弃陈规陋习、养成文明习惯，形成精致、细腻、整洁、有序的城市环境。积极推进中外合作办学，培育具有明显竞争力的名牌学校和品牌专业，加强各级医疗机构临床重点专科、学科建设，引进建设一批高层次专科医院，更大力度打造淮海经济区教育、医疗中心。抓好文化产业集聚区、创意街区、文博展区建设，丰富文化业态、产品和服务，加快建设文化强市。抓好街头绿地建设和道路绿化提升，实施五山公园、泉润公园等园林景观工程，打造云龙湖、潘安湖、吕梁山等生态片区，健全绿色生态网络体系。

着力构建新型城镇体系。在抓好5个中等城市建设的同时，深入开展试点创建，示范带动新型城镇化建设。选择8个镇（街道）开展新型中心镇试点，推动产业发展、基础设施、公共服务、城镇管理提档升级，构建县域经济社会发展副中心。科学选择21个试点，坚持政府引导、企业主体、市场化运作，加快创建一批产业、文化、旅游和社区功能叠加的特色小镇。启动新型社区（美丽乡村）建设“百村示范、千村提升”行动计划，打造一批规模适中、功能齐全、产业兴旺、环境优美的新型社区和美丽康居乡村。创新资金保障、土地利用、考核激励和城镇管理机制，采取PPP等多种模式引导社会资本参与，深化户籍制度改革，开展建设用地布局调整，市县联动实施土地综合整治，为城乡发展开辟更大空间。

加速发展壮大板块经济。鼓励各板块因地制宜培育特色产业，优化“一核五沿”生产力布局。繁荣发展城区经济，支持鼓楼、云龙、泉山区发展现代服务业和高新技术产业，推动铜山、贾汪区加快融入主城区、实现转型发展，提升徐州经济技术开发区、徐州高新技术开发区、新城区高端产业和高端要素集聚承载能力。继续加强“一县一策”分类指导，支持各县（市）坚持不懈抓招商、上项目，集中力量做大做强2-3个主导产业，提升县域在全市经济发展中的比重和贡献度，形成争先进位、竞相发展的生动局面。

（五）深化改革开放，营造活力迸发、动力强劲的良好氛围。坚定不移推进改革攻坚和开放转型，在全面深化改革中激发市场活力，在扩大双向开放中集聚发展要素，为全面振兴转型增添新动能、增创新优势。

打好全面深化改革攻坚战。深化供给侧结构性改革，一着不让推进煤炭、钢铁行业去产能，坚决淘汰落后产能，坚决禁止新增钢铁产能，坚决打击非法产能；千方百计抓好房地产调控，促进房地产市场平稳健康发展；以强化债务管理、扩大直接融资为抓手，稳妥审慎去杠杆；落实好减税、降费、降低要素成本等各项措施，打好降低实体经济企业成本“组合拳”；加快补齐基础设施、新型城镇化、扶贫攻坚等薄弱领域短板。深入推进“放管服”改革，抓好权力清单标准化建设，深化相对集中许可权改革；推进综合行政执法体制改革，健全完善执法监管制度体系；推动“红顶中介”与行政机关脱钩；继续开展事业单位分类改革。深化国企国资改革，加快形成有效制衡的公司法人治理结构、灵活高效的市场化经营机制，推动国有资本投资、运营公司改革试点。稳妥推进财税和金融体制改革，落实好税制改革各项政策，推动金融机构开展投贷联动，推进两权抵押贷款试点，加强金融风险防控。深化农村产权制度改革，明晰农村集体产权归属，完成农村土地承包经营权确权登记颁证，探索农户对土地承包权、宅基地使用权、集体收益分配权的自愿有偿退出机制。积极创建国家老工业城市和资源型城市产业转型升级示范区、沿东陇海线经济带双向开放改革试验区。

更高水平推进双向开放。积极学习、全面复制上海等自贸区改革经验，完善开放平台、开发园区、开放型经济体系。在招商引资上下功夫，牢牢抓住招商引资这条生命线，制定更具竞争力的扶持政策，突出领导招商、全产业链招商，重构招商队伍；办好第二十届投资洽谈会等系列重点招商活动，加大招商项目跟踪落实力度，确保引进3000万美元重大外资项目个数增长10%以上，力争引进一批超亿美元乃至10亿美元的重大项目；争取中建材薄膜太阳能电池、华信综合物流新能源等33项前期推进项目取得实质性进展，努力使一批招商成果转化为当年实施项目。在扩大出口上下功夫，做大做强工程机械、地坪地材、大蒜三大出口基地，稳定重点产品出口，扩大进出口总额。在平台建设上下功夫，抓好电子口岸建设、陆路口岸开放、航空口岸功能提升，争创徐州综合保税区，统筹推进新沂保税物流中心、国际邮件互换中心等海关特殊监管区建设，争取跨境贸易电子商务改革试点。在园区建设上下功夫，加快各类开发园区转型升级步伐，推动徐州经济技术开发区、高新区向高标准综合园区转型，支持沛县、邳州、新沂开发区争创国家级开发区，力争一批特色园区升格为省级开发园区。在对外合作上下功夫，全面落实国际产能和装备制造合作三年行动计划，推动企业更大步伐“走出去”。

（六）加强环境保护，走生态优先、绿色发展的新路子。本着对徐州发展、对人民群众高度负责的态度，以背水一战的决心、壮士断腕的勇气，全力开展“两减六治三提升”环保专项行动，认真落实中央环境保护督查整改要求，更加自觉、更大力度推进生态文明建设。

以“两减”为突破口，切实减少污染物源头排放。实施重点产业园区规划环评负面清单管理，严格控制项目准入门槛，严格限制高能耗高污染项目落地，坚决取缔“地条钢”、小焦化和违法违规钢铁产能，基本完成主城区钢铁、化工、有色金属冶炼、水泥等重污染企业搬迁改造，倒逼产业由重转轻、由旧转新、由低转高。减少煤炭消费总量，大力调整能源结构，推进煤电机组超低排放改造、非电行业全要素达标排放，对钢铁、水泥行业耗煤项目实施煤炭消费量减量替代；分类整治燃煤锅炉，禁止新建燃煤供热锅炉；大力发展清洁能源和清洁生产。减少落后化工产能，开展化工企业基本情况排查，坚决淘汰生产工艺和技术装备落后、达不到安全和环保要求的化工企业；清理并规范化工园区，禁止新增化工园区；推动化工企业入园进区，禁止园区外新建、扩建化工项目。

以“六治”为主抓手，加快解决突出环境问题。治理生活垃圾，推进垃圾源头减量和资源化利用，健全垃圾分类收集、中转和处理处置体系，加快建设餐厨废弃物处理厂和第二生活垃圾焚烧厂。治理黑臭水体，抓好城镇黑臭河道治理，完善城镇雨污分流管网和污水处理设施，加快城市污水处理厂提标改造；实施河塘清淤疏浚，构建健康水循环体系。治理畜禽养殖污染，依法划定畜禽养殖禁养区、限养区，加快建设规模化畜禽养殖场粪污收集、处理利用设施。治理挥发性有机物污染，开展化工企业泄漏检测与修复，完成重点化工园区和重点企业废气排放源整治；加快淘汰老旧车辆，全面推广新能源汽车；加强餐饮油烟污染控制和露天烧烤整治。治理采矿塌陷地，做好采空区地面塌陷隐患勘察、监测、预防，做好塌陷地沉稳区、工矿废弃地和采石宕口生态修复。治理环境隐患，实施重金属污染防治综合整治工程，落实土地污染防治行动计划，开展重点风险企业环境安全达标建设，加强秸秆禁烧和综合利用，防范和化解环境风险隐患。

以“三提升”为着力点，健全生态保护长效机制。提升生态保护水平，积极参与江淮生态大走廊建设，完成市级生态红线区域优化调整和国家、省级生态保护红线划定，落实河长制和水流产权确权，抓好国土绿化和湿地保护。提升环境经济政策调控水平，落实差别化的环境价格政策，全面推行排污权有偿使用和交易制度，完善生态补偿机制；强化建设用地批后监管，整体推进建设用地节约集约利用评价、工业用地调查以及低效产业用地再开发，争创全国土地节约集约利用模范市。提升环境执法监管水平，建立网格化监管执法责任体系，铁腕执法、铁面问责，严厉精准打击各类环境违法行为。

四、全面提升政府自身建设水平

紧紧围绕建设服务政府、法治政府和廉洁政府，进一步加强政府治理体系和治理能力建设，着力打造优质高效的政府服务环境、公平公正的法治环境、诚实守信的信用环境，不断提升政府的公信力执行力凝聚力。

注重科学施政，树立干事创业的政府形象。进一步匡正发展理念、提升发展境界，统筹处理好各方面的关系，努力实现遵循发展规律、市场规律、自然规律的科学发展。继续落实好市政府每周工作例会、重点工作定期调度、重大问题分层级协调解决等制度，加强绩效管理和政务督查，健全政府工作高效运行机制。不搞虚假政绩，不搞数字攀比，出实招、办实事、求实效，大力营造想干事、能干事、干成事的浓厚氛围。

坚持勤政为民，树立务实高效的政府形象。以更严更实的举措抓好机关作风建设，强化便民意识和服务意识，全力打通“中梗阻”，坚决纠正不作为、乱作为、慢作为，坚决惩处失职、渎职，坚决查处少数党员干部不负责任、不敢担当以及庸懒散拖等现象，切实提高政府办事效率。抓好简政放权和行政审批流程再造，提升“互联网+政务服务”水平，深入推进行政审批服务、公共资源交易、12345政府服务热线等平台建设，构建“1＋X”一体化管理运行的综合便民服务中心。办好人大议案、建议和政协提案，做到事事有回音、件件有着落。

加强依法行政，树立阳光法治的政府形象。牢固树立依法治市的理念，全面推进法治政府、法治市场、法治社会一体化建设，使法治成为徐州核心竞争力的重要标志。健全依法科学民主决策机制，完善政府法律顾问制度，深化政务公开。落实行政执法公示、重大行政执法决定法制审核、行政执法全程记录制度，促进严格规范公正文明执法。主动接受人大及其常委会的法律监督和工作监督，自觉接受政协民主监督，强化同民主党派的联系，重视公众监督、舆论监督，加强层级监督和专门监督，确保权力在法治轨道上正确运行。

全面从严治政，树立清正廉洁的政府形象。严格落实主体责任，认真履行“一岗双责”，扎实推进廉洁政治“三清示范区”建设。自觉遵守党的政治纪律和政治规矩，切实增强“四个意识”，坚定不移维护以习近平同志为核心的党中央权威，强化行政问责制度，确保有令即行、有禁即止。坚持不懈纠正“四风”，坚持用制度管权管人管事，加大行政监察和审计监督力度，严管公共资金，严管公共资源，严管国有资产资本。坚持无禁区、全覆盖、零容忍，保持反腐败斗争高压态势，严查发生在群众身边的不正之风和腐败行为，加快形成不敢腐、不能腐、不想腐的体制机制。

同志们，徐州的发展正处在继往开来的重要历史时期。让我们更加紧密地团结在以习近平同志为核心的党中央周围，高举中国特色社会主义伟大旗帜，在中共徐州市委的领导下，不忘初心、继续前进，同心同德、顽强拼搏，以优异成绩迎接党的十九大胜利召开！
